# Dairy Product Consumption and Oligo-Astheno-Teratozoospermia Risk: A Hospital-Based Case-Control Study in China

**DOI:** 10.3389/fnut.2021.742375

**Published:** 2021-12-21

**Authors:** Xiao-Bin Wang, Qi-Jun Wu, Ren-Hao Guo, Xu Leng, Qiang Du, Yu-Hong Zhao, Bo-Chen Pan

**Affiliations:** ^1^Center for Reproductive Medicine, Department of Obstetrics and Gynecology, Shengjing Hospital of China Medical University, Shenyang, China; ^2^Department of Clinical Epidemiology, Shengjing Hospital of China Medical University, Shenyang, China; ^3^Clinical Research Center, Shengjing Hospital of China Medical University, Shenyang, China

**Keywords:** association, case-control study, China, dairy product, oligo-astheno-teratozoospermia

## Abstract

**Background:** Researches on the association of dairy products consumption with Oligo-astheno-teratozoospermia (OAT) risk has been limited and controversial. Therefore, we aim to explore the aforementioned association among Chinese men.

**Methods:** A hospital-based case-control study was conducted in men consisting of 106 cases of OAT and 581 controls. Intakes of dairy products and their related nutrients were collected using a semi-quantitative food frequency questionnaire and semen quality was analyzed according to the World Health Organization guidelines. The daily intake of dairy products and their related nutrients was categorized into three groups with the lowest tertile serving as the reference category. Odds ratios (ORs) and 95% confidence intervals (CIs) of association between dairy intake and OTA risk were calculated by the multivariable logistic regression models.

**Results:** No significant association was found between total dairy (OR _T3vs.T1_ =1.53, 95% CI: 0.85–2.78), protein (OR _T3vs.T1_ =1.51, 95% CI: 0.84–2.75), or calcium (OR _T3vs.T1_ = 1.46, 95% CI: 0.81–2.66) and the OAT risk in the main findings. However, we observed a statistically significant positive association of dairy fat intake with OAT risk (OR _T3vs.T1_ =1.93, 95% CI: 1.06–3.58). The findings were consistent with the main results when we carried out subgroup analysis stratified by body mass index.

**Conclusion:** A significant positive association was found between dairy fat intake and the risk of OAT. Further large-scale prospective studies are required to confirm this finding.

## Introduction

Oligo-astheno-teratozoospermia (OAT) is one of the major causes of infertility in men (1). OAT is diagnosed if the sperm count and the percentage of motility and normal morphology are lower than the World Health Organization (WHO) reference value (2). A large sperm bank from China with 30,636 young men revealed that the percentage of progressively motile sperms, the percentage of normal sperm morphology, and sperm concentration decreased by 38, 20, and 31%, respectively during 2001 to 2015 (3). Infertility has been regarded as a rapidly emerging global public health issue during the recent decades (4). Notably, childlessness attributed to the male factors accounts for 20 to 70% (5). Numerous causative factors might be involved in the genesis of the disease, including environmental exposure (6), varicocele (7), older age (8), unhealthy lifestyle (9–11), and endocrine-disrupting chemicals (12). A large body of evidence reported that dietary pattern and nutrition were associated with sperm concentration and morphology (13–16). For example, lycopene and nut consumption was associated with better sperm morphology, while inverse association was found between adherence to the Western pattern (characterized by high intakes of french fries, processed meats, and snacks) and sperm concentration (13–15).

Dairy products and their related nutrients constitute a key part of our diet, and the relationship between intake of dairy products and their related nutrients and the public health problems has attracted great attention (17, 18). There are wide variety of dairy products including yogurt, cheese, whole milk, low-fat milk, and skim milk, some of which are rich in protein, fat, minerals, and vitamins (19, 20). Compared with developed countries, intake of dairy products among Chinese residents is relatively low (21). Previous studies explored the associations between intake of dairy products and male infertility but yielded inconsistent results or had the limitation of small sample size (22–26). For example, in Iran, a case-control study comparing 72 cases of asthenozoospermia with 169 controls of normozoospermia reported that dairy products consumption was unrelated to the asthenozoospermia risk (24). However, a case-control study with 30 cases of poor semen quality and 31 controls conducted in Spain observed negative associations between dairy products and semen quality (25).

As far as we know, to date, there is no study exploring the association of dairy products intake with OAT risk in China. Therefore, the present hospital-based case-control study involving 106 cases and 581 controls attempted to explore the associations of intake of total dairy products and their related nutrients including protein, calcium, and fat with the risk of OAT.

## Materials and Methods

### Design and Population

We designed a hospital-based case-control study, conducting from June, 2020 to December, 2020, which enrolled men who admitted to the infertility clinic of Shengjing Hospital of China Medical University. After the primary infertility exams, participants were divided into two groups according to the World Health Organization (WHO) laboratory manual for the examination and processing of human semen (27): (1) Cases (*n* = 118): men with OAT, which was defined as the concentration, the proportions of progressively motile, and morphologically normal spermatozoa below the reference values (<15 × 10^6^ of sperms/mL, <32% progressive motility, and <4% normal morphology); and (2) Controls (*n* = 612): men with normozoospermia from infertile couples (≥15 × 10^6^ of sperms/mL, ≥32% progressive motility, ≥40% total motility, and ≥4% normal morphology). Trained interviewers helped participants complete a validated 110-item food frequency questionnaire (FFQ). We excluded participants who did not complete the FFQ or had incomplete information and implausible total caloric intake (<800 or >4,200 kcal/d), and cases with a history of varicocele. Finally, 106 incident cases (90%) and 581 controls (95%) met all eligible criteria and were included in the final analysis. The study protocol was approved by the ethics committee of Shengjing Hospital of China Medical University (2017PS190K). All research participants signed written, informed consents.

### Semen Analysis

All participants were instructed to abstain from ejaculation for 3–7 days before semen collection. Semen samples were collected on-site by masturbation into a plastic container without using condoms or lubricants in a dedicated semen collection room. Before analysis, the specimen was liquefied for <60 min. Ejaculate volume and PH were directly measured. Semen parameters such as sperm concentration and motility including the percentage of each motility grade were tested with a WLJY9000 instrument. Sperm smear was stained with Papanicolaou method and observation of the sperm morphology was carried out with optical microscopy. Additionally, sperm DNA fragmentation was assessed through flow cytometry. The WHO criteria were used to define the semen quality parameters (27). All analyses were performed by an experienced technician and external quality control was performed throughout the study.

### Data Collection

Anthropometric parameters (height, weight, and waist circumference) were measured using a standard protocol. Additionally, age, television watching, computer using, smoking status (yes or no), alcohol drinking status (yes or no), tea drinking (yes or no), coffee drinking (yes or no), education (junior secondary or below, senior high school/technical secondary school, or junior college/university or above), and annual household income (<50, 50–100, or ≥100 thousand yuan) were obtained from the questionnaire. Anxiety and depressive symptoms were measured with GAD-7 (Generalized Anxiety Disorde-7) and PHQ-9 (Patient Health Questionnaire-9), respectively. The Metabolic equivalent (MET) of each activity was subsequently multiplied by the frequency and duration of physical activity to calculate total physical activity in MET hours per day (MET-h/d) (28).

### Dietary Assessment

We used the validated 110-item FFQ to estimate average daily intake of food and nutrients based on usual dietary consumption over the year before the interview. Participants were asked to report how often, on average, they had consumed each type of food. Seven frequency options were set for selection, including “more than two times per day,” “1–2 times per day,” “4–6 times per week,” “2–3 times per week,” “one time per week,” “2–3 times per month,” or “never.” Nutrient intakes for each food were estimated using the Chinese Food Composition Tables (29). After completion, the auditor verified and checked the completeness of the FFQ. If the options were missing or unclear, we would contact participants and make corrections in time. The reproducibility and validity of the questionnaire used in the current study were similar to those of previous versions with 111-item FFQ (30).

### Statistical Analysis

The normality of all continuous variables was evaluated through the Kolmogorov-Smirnov statistic. The Student's *t*-tests and the chi-square tests were used for comparison of continuous variables and categorical variables between the two groups, respectively. Results of continuous variables were expressed as means ± standard deviation (SD) and categorical variables were expressed as count with percentage. We divided the daily intake of dairy products and their related nutrients (protein, fat, and calcium) into tertiles based on the distribution of control subjects, and the lowest tertile was used as the reference category. The principal component method for factor analysis was used to derive potential dietary patterns (31). An unconditional multivariable logistic regression model was used to calculate odds ratios (ORs) and the corresponding 95% confidence intervals (CIs). We only adjusted for age (years) in the first model and further adjusted for body mass index (BMI) (kg/m^2^), total energy intake (kcal), smoking status (no/yes), abstinence time (days), household income (RMB thousand yuan), and dietary patterns except those for dairy products in the second model. In addition, we performed subgroup analysis stratified by BMI to further estimate the adjusted risk between intake of dairy products and their related nutrients and the OAT risk. *P* values for trend were calculated by using the median values of the dairy products and their related nutrients categories, and modeling the variable as a continuous variable. Statistical significance was set at *p* < 0.05 and based on the two-sided test. All analyses were carried out in SAS version 9.4 (SAS Institute Inc., Cary, NC, USA).

## Result

The baseline characteristics of the study subjects are shown in Table 1. The main analysis consisted of 106 OAT cases and 581 controls. Compared with controls, OAT cases had significantly higher PHQ-9 score and GAD-7 score. Conversely, compared with controls, OAT cases had significantly lower sperm concentration, total sperm count, progressive motility, total motility, and percentage of normal sperm morphology as well as shorter abstinence time. Furthermore, the mean egg intake in cases was significantly higher than that in controls.

**Table 1:** General characteristics of participants in a case-control study of oligo-astheno-teratozoospermia.

**Characteristics**	**Case**	**Control**	***P* value**
No. of participants	106	581	
Age (years)	33.01 ± 4.71	32.11 ± 4.50	0.06
Body mass index (kg/m^2^)	26.04 ± 4.15	26.25 ± 4.55	0.66
Physical activity (MET/hours/week)	169.90 ± 109.40	165.90 ± 101.80	0.71
Television watching (hours/week)	5.49 ± 8.23	6.19 ± 8.01	0.41
Computer using (hours/week)	23.74 ± 15.09	24.90 ± 15.07	0.47
PHQ-9 (score)	4.04 ± 4.16	2.71 ± 3.58	<0.05
GAD-7 (score)	3.15 ± 4.04	1.75 ± 2.75	<0.05
Abstinence time (days)	3.98 ± 1.35	4.28 ± 1.39	<0.05
**Semen parameters**			
Ejaculate volume (ml)	3.20 ± 1.72	3.45 ± 1.26	0.16
Sperm concentration (10^6^/ml)	9.71 ± 20.97	71.10 ± 39.88	<0.05
Total sperm count (10^6^/ml)	26.19 ± 53.38	232.20 ± 133.50	<0.05
Progressive motility (%)	14.04 ± 10.62	44.58 ± 9.34	<0.05
Total motility (%)	17.61 ± 12.91	54.94 ± 11.35	<0.05
Normal sperm morphology (%)	1.35 ± 1.38	6.68 ± 2.73	<0.05
**Diet**			
Meat (g/day)	98.39 ± 46.11	106.70 ± 49.02	0.11
Eggs (g/day)	44.44 ± 35.68	32.22 ± 25.26	<0.05
Fish and seafood (g/day)	23.86 ± 18.58	22.84 ± 20.49	0.63
Beans and bean products (g/day)	107.60 ± 91.22	92.10 ± 82.43	0.08
Vegetables (g/day)	218.30 ± 157.90	194.00 ± 130.20	0.14
Fruits (g/day)	169.20 ± 163.40	167.20 ± 171.60	0.91
Total energy intake (kcal/day)	1876.20 ± 558.70	1769.10 ± 562.00	0.07
**Smoke status (** ***n*** **, %)**			1.00
No	50 (47.17)	274 (47.16)	
Yes	56 (52.83)	307 (52.84)	
**Alcohol intake (** ***n*** **, %)**			0.94
No	60 (56.60)	331 (56.97)	
Yes	46 (43.40)	250 (43.03)	
**Tea drinking (** ***n*** **, %)**			0.57
No	68 (64.15)	389 (66.95)	
Yes	38 (35.85)	192 (33.05)	
**Coffee drinking (** ***n*** **, %)**			0.78
No	96 (90.57)	531 (91.39)	
Yes	10 (9.43)	50 (8.61)	
**Educational level (** ***n*** **, %)**			0.34
Junior secondary or below	24 (22.65)	141 (24.27)	
Senior high school/technical secondary school	10 (9.43)	82 (14.11)	
Junior college/university or above	72 (67.92)	358 (61.62)	
**Annual family income (RMB thousand yuan), (** ***n*** **, %)**			0.36
<50	23 (21.70)	94 (16.18)	
50 – <100	40 (37.74)	226 (38.90)	
≥100	43 (40.56)	261 (44.92)	

*MET, metabolic equivalent; PHQ-9, patient health questionnaire-9; GAD-7, generalized anxiety disorder-7.*

*All continuous variables are shown as the mean and standard deviation. P values were determined with Student's t test and chi-square test.*

*All statistical tests are two sided*.

The associations between intake of dairy product and their related nutrients and the risk of OAT are presented in Table 2. After adjusting for potential confounders, a comparison of the highest tertile with the lowest tertile in the intake of dairy fat showed that higher intake of dairy fat was associated with an increased risk of OAT in model 1 (OR _T3vs.T1_ =1.73, 95% CI: 1.02–3.00) and model 2 (OR _T3vs.T1_ =1.93, 95% CI: 1.06–3.58). However, there were no significant associations between intake of total dairy products and their other related nutrients and the risk of OAT.

**Table 2:** Adjusted ORs and 95% CIs for oligo-astheno-teratozoospermia by intake of dairy products and their related nutrients.

**Variables**	**Cases** **(*N* = 106)**	**Controls** **(*N* = 581)**	**Model 1^**b**^**	**Model 2^**c**^**
**Total dairy**^**a**^ **(g/day)**
T1 (<31.16)	27	176	1.00 (Ref)	1.00 (Ref)
T2 (31.16 – <135.66)	35	205	1.11 (0.64–1.91)	1.14 (0.65–2.01)
T3 (≥135.66)	44	200	1.44 (0.86–2.45)	1.53 (0.85–2.78)
*P* for trend			0.14	0.14
**Protein (g/day)**
T1 (<0.95)	27	173	1.00 (Ref)	1.00 (Ref)
T2 (0.95 – <4.16)	35	210	1.06 (0.62–1.84)	1.09 (0.62–1.92)
T3 (≥4.16)	44	198	1.43 (0.85–2.44)	1.51 (0.84–2.75)
*P* for trend			0.13	0.14
**Fat (g/day)**
T1 (<0.97)	24	180	1.00 (Ref)	1.00 (Ref)
T2 (0.97 – <3.64)	37	205	1.36 (0.78–2.38)	1.51 (0.85–2.71)
T3 (≥3.64)	45	196	1.73 (1.02–3.00)	1.93 (1.06–3.58)
*P* for trend			0.05	0.05
**Calcium (mg/day)**
T1 (<36.44)	28	180	1.00 (Ref)	1.00 (Ref)
T2 (36.44 – <158.65)	36	201	1.14 (0.67–1.96)	1.15 (0.66–2.02)
T3 (≥158.65)	42	200	1.37 (0.82–2.32)	1.46 (0.81–2.66)
*P* for trend			0.24	0.20

*CI, confidence interval; OR, odds ratio; T, tertiles; Ref, reference.*

*^a^Total dairy includes whole milk, skim/low-fat milk, cheese, and yogurt.*

*^b^Model 1: adjusted for age.*

*^c^Model 2: adjusted for age, body mass index, smoking status, household income, total energy intake, abstinence time, and dietary patterns except those for dairy products*.

Table 3 shows the association of total dairy products and their related nutrients with the risk of OAT stratified by BMI. However, no significant associations were observed between the total dairy products and their other related nutrients intake and the risk of OAT after adjusting for the aforementioned confounding factors. Similarly, no significant interactions were observed between the total dairy product and their other related nutrients intake and BMI.

**Table 3:** Adjusted ORs and 95% CIs for oligo-astheno-teratozoospermia by intake of dairy products and their related nutrients, stratified by body mass index.

**Variables**		**Cases (*N* = 106)**	**Controls (*N* = 581)**	**OR (95% CI)^**a**^**	**OR (95% CI)^**b**^**	***P* for interaction**
Total dairy^c^	**<25**					0.09
(g/day)	T1 (<31.16)	14	78	1.00 (Ref)	1.00 (Ref)	
	T2 (31.16 – <135.66)	13	78	0.96 (0.42–2.20)	0.90 (0.37–2.18)	
	T3 (≥135.66)	14	83	0.98 (0.43–2.22)	1.05 (0.40–2.73)	
	*P* for trend			0.97	0.86	
	**≥25**	
	T1 (<31.16)	13	98	1.00 (Ref)	1.00 (Ref)	
	T2 (31.16 – <135.66)	22	127	1.30 (0.63–2.77)	1.25 (0.59–2.73)	
	T3 (≥135.66)	30	117	1.93 (0.97–4.02)	1.84 (0.85–4.14)	
	*P* for trend			0.05	0.11	
Protein	**<25**					0.13
(g/day)	T1 (<0.95)	14	78	1.00 (Ref)	1.00 (Ref)	
	T2 (0.95 – <4.16)	13	79	0.95 (0.41–2.18)	0.90 (0.37–2.17)	
	T3 (≥4.16)	14	82	0.99 (0.43–2.24)	1.06 (0.40–2.76)	
	*P* for trend			1.00	0.84	
	**≥25**	
	T1 (<1.00)	17	112	1.00 (Ref)	1.00 (Ref)	
	T2 (1.00 – <4.16)	18	114	1.03 (0.50–2.12)	0.99 (0.47–2.09)	
	T3 (≥4.16)	30	116	1.70 (0.90–3.31)	1.60 (0.77–3.40)	
	*P* for trend			0.07	0.15	
Fat	**<25**					0.21
(g/day)	T1 (<0.84)	11	78	1.00 (Ref)	1.00 (Ref)	
	T2 (0.84 – <3.64)	15	81	1.39 (0.60–3.33)	1.51 (0.61–3.90)	
	T3 (≥3.64)	15	80	1.39 (0.60–3.32)	1.66 (0.61–4.60)	
	*P* for trend			0.58	0.44	
	**≥25**	
	T1 (<0.97)	12	98	1.00 (Ref)	1.00 (Ref)	
	T2 (0.97 – <3.64)	23	128	1.46 (0.70–3.17)	1.48 (0.69–3.28)	
	T3 (≥3.64)	30	116	2.11 (1.05–4.48)	2.06 (0.94–4.71)	
	*P* for trend			<0.05	0.08	
Calcium	**<25**					0.07
(mg/day)	T1 (<35.75)	14	78	1.00 (Ref)	1.00 (Ref)	
	T2 (35.75 – <158.65)	13	79	0.94 (0.41–2.15)	0.87 (0.36–2.11)	
	T3 (≥158.65)	14	82	1.00 (0.44–2.27)	1.10 (0.42–2.90)	
	*P* for trend			0.97	0.76	
	**≥25**	
	T1 (<36.44)	13	98	1.00 (Ref)	1.00 (Ref)	
	T2 (36.44 – <158.65)	24	126	1.43 (0.70–3.02)	1.34 (0.64–2.90)	
	T3 (≥158.65)	28	118	1.79 (0.89–3.74)	1.70 (0.78–3.83)	
	*P* for trend			0.13	0.20	

*CI, confidence interval; OR, odds ratio; T, tertiles; Ref, reference.*

*^a^OR (95%CI): adjusted for age.*

*^b^OR (95%CI): adjusted for age, body mass index, smoking status, household income, total energy intake, abstinence time, and dietary patterns except those for dairy products.*

*^c^Total dairy includes whole milk, skim/low-fat milk, cheese, and yogurt*.

## Discussion

In the present study, our results indicated that intake of dairy fat was positively associated with the risk of OAT. However, no significant associations were observed between total dairy products, their protein and calcium content and the risk of OAT in the main and subgroup analyses.

Previous studies investigated the association between dairy products and the risk of male infertility, but reported inconsistent results. For instance, a longitudinal study recruiting 255 men in a fertility clinic in USA found that intake of total dairy products (including cheese, cream, ice cream, and whole milk) was unrelated to semen quality parameters (22). Similarly, no significant association was observed between dairy product intake and semen quality in a cross-sectional study in the Netherlands (26). Additionally, a case-control study involving 72 asthenozoospermia cases and 169 controls conducted in Iran indicated that intake of total dairy product was uncorrelated with asthenozoospermia risk (24). In contrast, a case-control study with 31 controls and 30 cases of OAT conducted in Spain revealed the cases had higher intakes (means of intake frequencies = 3.3) of dairy products compared with controls (means of intake frequencies = 2.7) (25). Besides, a cross-sectional study carried out in USA with 189 young college students also showed that, as the consumption of dairy products increased, the risk of abnormal sperm morphology became higher (23). We speculate that the inconsistency with our results may be attributed to differences in the study design, age of population, and sample size. For example, compared with our study (an average age of 32.68 years), the population in that cross-sectional study (an average age of 19.7 years) was relatively younger (23).

Up to date, several studies have explored the mechanism of dairy products and OAT. A previous study reported that men who consumed milk obtained from pregnant cows containing natural estrogens had significantly increased serum estrone, progesterone concentrations and decreased serum luteinizing hormone, follicle-stimulating hormone, and testosterone (32, 33), which might partially explain the decrease in sperm production. Additionally, spermatogenesis is a process of active cell division requiring insulin, and Leydig cell insulin receptors can be bound and activated by insulin growth factor-1 (IGF-1) whose circulating concentrations were also significantly and positively associated with dairy products (34, 35). In line with our observation that dairy fat can increase the risk of OAT, it is interesting to note that one study reported low-fat dairy intake was positively related to sperm concentration and progressive motility (23). Another study reported that consumption of low-fat and skimmed milk was associated with increased circulating levels of insulin, probably through increasing the levels of IGF-1; and animal studies indicated that subcutaneous insulin administration may increase sperm motility and concentration (36). In addition, several studies have found that a high-fat diet may reshape the ecosystem of the gut microbiome (37, 38), leading to abnormal gene expression in the testes, low sperm motility, and defective sperm production (39).

There are several strengths in our study. Firstly, we used a validated FFQ to explore the relationship between dairy products and their related nutrients consumption and the risk of OAT. Secondly, compared with previous studies of the same type (25), the relatively larger sample sizes with cases (*n* = 106) and controls (*n* = 581) of this study provided more reliable results. Thirdly, the participation rate of both groups (90% in cases and 95% in controls) were high in the present study.

Meanwhile, the present study has some limitations. Firstly, because of the case-control design, selection and recall biases are inevitable. However, in order to reduce such bias, well-trained investigators were used in collecting the dietary information. In addition, we collected the frequency rather than consumption content for dairy products via a semi-quantitative FFQ, and food photographs were provided to assist participants with the quantification of dairy product intake, which could help reduce the impact of recall bias. Moreover, as the dairy products were collected in the last 12 months before the diagnosis, a temporal relation between dairy products and their related nutrients and OAT was a concern, because having a higher intake of diet in the past at a younger age may have an effect on semen quality (40, 41). Besides, in order to increase the comparability of case and control, we recruited controls in the same clinical setting in the infertility clinic. Secondly, owing to the relatively low consumption of dairy product subtypes (e.g., whole milk, skim/low fat milk, cheese, and yogurt), we could not evaluate the association between dairy product subtypes and the OAT risk. Thirdly, although we have adjusted for many confounding factors, residual confounding remains a possible concern for our results, together with potential unmeasured confounders, such as genetic factors. Matching cases and controls according to BMI values would be ideal. However, overmatching problem would be a concern, which might led to loss of efficiency as the matching effect could narrow the exposure range. Fourthly, since the present study was a single-center research, the findings might not be perfectly generalized to other populations. Therefore, future studies with a multi-center approach and a larger sample size would be desirable. Finally, as it was a case-control study, we could not determine causality of the observed associations. Further large prospective cohort studies with assessment of more variety of dairy products and well-designed randomized controlled trials are needed to assess the association between dairy products intake and OAT risk.

In conclusion, consumption of dairy fat was associated with higher risk of OAT. However, due to the limitation of low intake of dairy product subtype as well as potential residual confounding, further research is warranted to confirm our findings.

## Data Availability Statement

The raw data supporting the conclusions of this article will be made available by the authors, without undue reservation.

## Ethics Statement

The studies involving human participants were reviewed and approved by the Ethics Committee of Shengjing Hospital of China Medical University. The patients/participants provided their written informed consent to participate in this study.

## Author Contributions

Q-JW and B-CP conceived the study. X-BW, Q-JW, and B-CP contributed to the design and interpreted the data. X-BW, R-HG, XL, and QD collected the data. X-BW and Q-JW cleaned the data, checked the discrepancy, and analyzed the data. All authors interpreted the data, read the manuscript, and approved the final vision.

## Funding

This work was supported by the National Key R&D Program of China No. 2017YFC0907403 to Y-HZ and Shengjing Hospital Clinical Research Project No. M0071 to B-CP.

## Conflict of Interest

The authors declare that the research was conducted in the absence of any commercial or financial relationships that could be construed as a potential conflict of interest.

## Publisher's Note

All claims expressed in this article are solely those of the authors and do not necessarily represent those of their affiliated organizations, or those of the publisher, the editors and the reviewers. Any product that may be evaluated in this article, or claim that may be made by its manufacturer, is not guaranteed or endorsed by the publisher.
